# Clinical and ethical considerations for pediatric cardiac xenotransplantation candidates

**DOI:** 10.3389/frtra.2026.1999444

**Published:** 2026-10-27

**Authors:** Eli Contorno, Sho Takemoto, Herra Javed, Sujay Sreenivasan, SunYoung Kim, Michael Schmitz, Taufiek Rajab

**Affiliations:** 1 Department of Cardiovascular Surgery, Arkansas Children’s Hospital, Little Rock, AR, United States; 2 Center for Transplantation Science, Massachusetts General Hospital and Harvard Medical School, Boston, MA, United States; 3 Department of Cardiovascular Anesthesiology, Arkansas Children’s Hospital, Little Rock, AR, United States

**Keywords:** congenital heart disease, fontan circulation, partial heart transplantation, pediatric heart transplantation, single-ventricle palliation, transplant ethics, xenotransplantation

## Abstract

Children with complex congenital heart disease face substantial mortality and morbidity, while access to heart transplantation remains constrained by donor scarcity. Limited mechanical circulatory support options further complicate bridging to transplantation, particularly in small infants. Cardiac xenotransplantation could provide a source of size-appropriate grafts, but its potential benefit depends upon many patient factors. This review examines clinical outcomes, pediatric preclinical evidence, and ethical considerations relevant to candidate selection, with particular attention to the single-ventricle pathway. Gene-edited porcine hearts have supported pediatric-sized nonhuman primates for months, with survival beyond 24 months in one recipient and a proof-of-concept transition to allotransplantation. Initial use as a bridge to allotransplantation may be most defensible in selected children with a poor prognosis under available palliation or mechanical support who could benefit from transplantation. Partial-heart xenotransplantation offers a separate potential application for living valve replacement. Ethical evaluation must address parental permission, the child's developing autonomy, and lifelong infection surveillance. Clinical translation requires evidence and safeguards matched to the proposed recipient population and a credible plan for subsequent treatment if xenograft function fails.

## 1 Introduction

Heart transplantation is an established treatment for selected children with end-stage heart failure or severe congenital heart disease (CHD) not amenable to conventional surgical management. CHD constitutes the predominant diagnostic group among infant heart transplant recipients, accounting for 61% of infant transplants in U.S. registry data from the 2010s (1). CHD and infant age have also been identified as independent risk factors for mortality while awaiting transplantation (2). In 2024, pretransplant mortality among U.S. candidates younger than 1 year was 17.7 deaths per 100 patient-years (3). The scarcity of size-appropriate donor hearts and more limited options for durable mechanical circulatory support (MCS) further constrain access to transplantation in this population (4, 5).

Cardiac xenotransplantation has attracted particular interest in infants and children with complex CHD, especially those with single-ventricle physiology. A readily available, size-appropriate xenograft could address both donor scarcity and the limited options for bridging these patients to allotransplantation. In patients with single-ventricle heart disease, the potential role of a xenograft extends beyond organ availability: Xenotransplantation could provide an alternative to further staged palliation in select high-risk patients or serve as a bridge to allotransplantation when conventional palliative strategies fail (6, 7).

In kidney transplantation, gene-edited (GE) porcine xenografts have progressed from single-patient expanded-access procedures (8) to prospective multicenter investigation in the ongoing EXPAND trial (NCT06878560), conducted under a U.S. Food and Drug Administration-cleared Investigational New Drug application (9, 10). In cardiac xenotransplantation, two adult recipients who were not candidates for conventional advanced heart-failure therapies received porcine hearts with 10 genetic modifications under expanded-access authorization. The xenografts initially provided life-supporting function, but both failed; the recipients survived for 60 and 40 days, respectively (11–13). Evidence directly applicable to children remains limited. Preclinical studies have generally addressed immunologic and physiologic barriers rather than pediatric-specific characteristics or uses, and human decedent studies have involved adults (14).

Although clinical or human-decedent transplantation of GE porcine hearts in children has not yet been reported, dedicated pediatric-sized nonhuman-primate models have achieved months-long xenograft survival, including survival beyond 24 months and proof-of-concept transition from xenotransplantation to allotransplantation (15). In a cohort of 15 orthotopic cardiac xenotransplantations, four died within 24 h of surgery, with eight surviving over 1-month. Among those surviving at least 1-month, median survival was 193 days. These findings support further investigation in pre-clinical models. Their clinical relevance depends on whether the anticipated benefit can justify the risks of xenotransplantation relative to established palliation, MCS, and allotransplantation. These questions are further complicated by pediatric-specific ethical issues surrounding recipient selection, parental authorization, future patient autonomy, and long-term surveillance (16).

This review examines the clinical and preclinical evidence and ethical considerations relevant to candidate selection for pediatric cardiac xenotransplantation, with particular attention to patients with single-ventricle physiology, the timing of xenotransplantation relative to staged palliation, and its potential role as a bridge to allotransplantation.

## 2 Single ventricle palliation and the role of heart transplantation

Staged palliation remains standard treatment for many forms of single-ventricle CHD, including hypoplastic left heart syndrome. The Norwood operation and subsequent Stage II and Fontan procedures have extended survival for conditions previously associated with high mortality in early infancy (17). Nevertheless, substantial mortality persists and morbidity accumulates. In the Pediatric Heart Network Single Ventricle Reconstruction Trial, transplant-free survival was 59% and 54% at 12 years in the right ventricle-to-pulmonary artery shunt and modified Blalock-Taussig-Thomas shunt groups, respectively (17). At 16-year follow-up, 87% of participants had experienced death, transplantation, or major morbidity, including severe or moderate impairments in adaptive behavior or quality of life, plastic bronchitis or protein-losing enteropathy, or right ventricular dysfunction (ejection fraction <45%) (18). Some patients cannot progress successfully through staged palliation, and heart transplantation may become an alternative to Fontan completion with progressive systemic ventricular dysfunction, significant atrioventricular-valve regurgitation, persistent cyanosis, unfavorable pulmonary vascular or anatomic conditions, or other barriers to a satisfactory Fontan circulation (19, 20).

Among patients who reach Fontan completion, medium-term survival is generally favorable. The Korean Fontan Registry reported overall survival after Fontan completion of approximately 92% at 10 years, 87% at 20 years, and 74% at 30 years (21). A nationwide Danish cohort reported 87% overall survival at 30 years (22). However, late complications—including ventricular dysfunction, atrial arrhythmias, Fontan-associated liver disease, protein-losing enteropathy and other lymphatic complications, and reduced exercise capacity—remain important sources of morbidity and may ultimately lead to Fontan failure (23–25).

Heart transplantation is a definitive treatment for selected patients with failing Fontan physiology, with outcomes associated with the recipient's condition at transplantation. In a multicenter U.S. cohort of 241 pediatric Fontan recipients, overall post-transplant survival was 89% at 1 year and 81% at 5 years; among patients without clinical decompensation, survival was 92% and 88%, respectively, compared with 83% and 74% among decompensated patients (26). Decompensation included need for extracorporeal membrane oxygenation, mechanical ventilation, and induced paralysis, as well as hepatic or renal dysfunction and dependence on parenteral nutrition. Decompensation was independently associated with worse post-transplant survival, supporting timely referral and assessment before advanced deterioration. The favorable outcomes of transplantation before advanced Fontan decompensation also raise the question of whether transplantation earlier in the single-ventricle pathway could avoid the cumulative burden of staged palliation.

Primary neonatal transplantation has historically provided an alternative to staged reconstruction for severe single-ventricle CHD. In a single-center series of 104 neonatal heart transplants, 78% of recipients had hypoplastic left heart syndrome or a related variant, with survival of 74% at 10 years and 56% at 25 years (27). However, neonatal heart transplantation remains uncommon in the United States (28), constrained by scarce size-matched donors and substantial waitlist mortality. An analysis of the Pediatric Heart Transplant Society registry recorded 35 waitlist deaths among 165 patients (21.2%) with single-ventricle CHD listed between 2010 and 2019 before any cardiac surgery (29). As palliative outcomes improved and donor availability remained limited, primary transplantation became less common; most infants with CHD undergoing transplantation now have prior cardiac surgery (4). Although evidence does not establish primary allotransplantation as superior to contemporary palliation, historical experience demonstrates durable survival after early replacement of the single-ventricle circulation when a suitable donor is available. Importantly, the outcomes reported here involve patients with heterogeneous characteristics and disease severity across different clinical eras and should not be compared directly. Fontan and post-transplant survival describe outcomes among patients who survived to those procedures, whereas the Single Ventricle Reconstruction trial estimates include death or transplantation from the Norwood operation onward. Waitlist mortality reflects the risk of death among listed patients before transplantation.

## 3 Preclinical evidence and pediatric considerations

Preclinical orthotopic cardiac xenotransplantation studies have achieved months-long survival of life-supporting xenografts in pediatric-sized baboons weighing 4.5–9.5 kg. The 15-recipient series used four different donor genetic constructs: two GGTA1-knockout with different human transgenes (*n* = 2, hCD55; *n* = 2, hCD46/hTBM), along with two different gene backgrounds with shared triple knockout, inactivated porcine endogenous retroviruses, and humans transgenes (hCD46/hCD55/hTBM/hEPCR/hCD47) donor backgrounds with additional varied human transgenes (*n* = 5, hTFPI/hHLA-E/hB2M; *n* = 6, hHO-1/hA20) (15). Induction therapy consisted of anti-thymocyte globulin, rituximab, methylprednisolone, and C1-esterase inhibition. Maintenance immunosuppression centered on costimulation blockade and rapamycin, with anti-CD40 replaced by anti-CD154 beginning with the fifth experiment. Etanercept was used in the first four experiments and tocilizumab from the tenth onward. In the initial 11 recipients, 6 (55%) survived more than 2 months (30). Following early graft dysfunction with University of Wisconsin solution, preservation was changed to modified del Nido cardioplegia with efforts to minimize graft ischemic time (15, 31). Chest drainage was also extended from removal at extubation to continued chest tube drainage for the first 10 postoperative days following early deaths from pulmonary edema and pleural effusions (30). In the expanded series of 15 recipients, median survival was 41 days; 8 (53%) survived more than 1 month, 6 exceeded 3 months, and the longest survivor exceeded 24 months (15). The longest survivor received anti-CD154-based immunosuppression but preceded the introduction of tocilizumab and prolonged pleural drainage. Reasons for early (< 1-month, *n* = 7) mortality included primary xenograft dysfunction (*n* = 1), mixed antibody-mediated and cellular rejection (*n* = 1), technical errors (*n* = 2), and systemic inflammatory xenograft response (*n* = 3). The contribution of individual protocol modifications and the reproducibility of prolonged cardiac xenograft support remain to be established (15). This report also included three baboons selected to undergo transition to allotransplantation following orthotopic cardiac xenotransplantation (15). One animal that had received a xenograft from the donor group additionally expressing hTFPI/hHLA-E/hB2M transitioned to allotransplantation 133 days after xenotransplantation and survived for 105 days post-allotransplant. Maintenance immunosuppression for this recipient initially comprised tacrolimus and mycophenolate mofetil, but suspected rejection subsequently required pulse corticosteroids, anti-CD20 and additional anti-CD154 therapy, with mycophenolate replaced by rapamycin. The animal was euthanized because of clinical deterioration, and necropsy demonstrated mixed antibody-mediated and cellular rejection. Another animal was euthanized intraoperatively following difficulties with pulmonary artery reconstruction; the third developed acute respiratory failure during induction therapy and was euthanized before the planned allotransplantation.

One concern when using a xenograft as a bridge is whether antibodies elicited against pig antigens could cross-react with human antigens and compromise subsequent allotransplantation. However, available preclinical evidence suggests that sensitization to pig antigens may not increase alloantibody responses (32). In the pediatric-sized model, no evidence of significant xeno- or allosensitization was reported during xenograft support (15). After allotransplantation, however, both anti-pig and anti-baboon IgG and IgM increased in the recipient who developed allograft rejection. The authors proposed that insufficient immunosuppression at transition and rejection of retained porcine inferior vena cava tissue, triggering systemic inflammation, contributed to this outcome. Whether antibody cross-reactivity contributed to the allograft rejection remains uncertain and warrants further investigation.

Human infancy is characterized by a distinct humoral immune profile that may be relevant to xenotransplantation. Natural cytotoxic anti-non-Gal antibodies were absent or present at low levels in most infants during the first year of life (33). Similarly, preformed IgM and IgG binding to triple-knockout pig red blood cells is uncommon in infants; in one study, no antibody binding was detected in 10 infants after Stage I Norwood palliation (34). Clinical experience with ABO-incompatible heart transplantation provides additional evidence of age-dependent B-cell immune plasticity. Donor-specific B-cell tolerance can develop after ABO-incompatible transplantation during infancy (35), and multicenter data have shown graft survival and other major outcomes comparable to those after ABO-compatible transplantation (36). These observations support investigation into whether the immune characteristics of infancy confer an advantage in cardiac xenograft acceptance and survival.

Whole-heart xenotransplantation in children must provide a graft whose size and function remain appropriate as the recipient grows. Excessive xenograft growth has been observed after orthotopic pig-to-baboon transplantation, and growth hormone receptor knockout substantially reduced graft hypertrophy for more than 6 months; however, physiologic mismatch persisted (37). Whether a growth-restricted porcine heart can maintain appropriate size and function as an infant recipient undergoes somatic growth remains unknown. Immunosuppression presents a separate challenge. Current nonhuman-primate protocols rely on lymphocyte-depleting induction, B-cell depletion, rapamycin, and CD40/CD154 costimulation blockade, but the intensity and long-term safety of these regimens in infants have not been established (6, 15, 38). Adult clinical experience further shows that extensive donor gene editing and costimulation blockade do not eliminate endothelial and antibody-mediated graft injury (12, 13).

Partial-heart xenotransplantation offers a separate approach to pediatric valve replacement. Valve repair is preferred when feasible, but complex congenital anatomy can limit reparability and durability, particularly in neonates and infants; recurrence and reintervention remain important problems (39). When repair is not feasible or fails, replacement is challenging because conventional prosthetic and biologic valves do not grow with the child and small annular dimensions restrict options. Children may require repeated interventions as valves are outgrown or deteriorate (40–43). Partial-heart allotransplantation addresses this limitation with viable valve tissue capable of adaptive growth. In a 19-patient series spanning ages two days to 34 years, detailed growth assessment focused on the initial nine infants. From early postoperative echocardiography to the latest follow-up, median annular diameters increased from 7 to 14 mm in the aortic position and from 9 to 17 mm in the pulmonary position, tracking along appropriate z scores; leaflet lengths also increased in all nine infants, supporting true tissue growth. Median follow-up for the overall cohort was 26.4 weeks, with the longest follow-up extending to 153 weeks (44). This procedure remains dependent on suitable human donor tissue. A porcine source could provide size-matched living valves without this constraint. Partial-heart xenografts may present a lower immunologic barrier than whole hearts because less donor tissue is transplanted and valve tissue appears relatively resistant to alloimmune injury, although reduced xenograft immunogenicity remains unproven (45).

Preclinical studies of partial-heart xenotransplantation remain at an early stage: a GE porcine right-ventricle-to-pulmonary-artery conduit implanted in an infant baboon maintained valve function without stenosis or regurgitation at 5 weeks of follow-up (46). One proposed strategy is to use a living xenogeneic valve during early childhood and replace it with an adult-sized conventional prosthesis once somatic growth permits, thereby limiting the period during which immunosuppression is required (40). Further studies will help determine whether this strategy can provide durable valve function and appropriate graft growth while limiting the burden of immunosuppression.

## 4 Timing and candidate selection across the single ventricle pathway

The clinical rationale for cardiac xenotransplantation changes across the stages of single-ventricle palliation. Existing proposals have generally favored its initial use as a bridge to allotransplantation rather than destination therapy, with particular emphasis on high-risk neonates and infants before or after Stage I palliation (6, 7). Candidate selection also requires a reasonable prospect of benefit from transplantation and a feasible route to subsequent allotransplantation. Illustrative candidates include children with severe ventricular dysfunction or significant atrioventricular valve regurgitation that limits further palliation, provided that extracardiac organ function is preserved or potentially recoverable and allotransplantation remains feasible. Table 1 summarizes these stage-specific considerations.

**Table 1:** Potential roles and unresolved risks of cardiac xenotransplantation across the single-ventricle pathway. All proposed roles concern selected transplant-eligible children for whom an allograft is unavailable and conventional palliation or mechanical support cannot provide an adequate bridge to allotransplantation.

Patient population and proposed role	Potential advantages	Disadvantages and unresolved risks
**Before Stage I palliation** High-risk neonates unlikely to survive Stage I palliation or the allograft waitlist who lack a durable mechanical bridge.	A size-appropriate GE donor could permit timely transplantation. May avoid further palliative operations and cumulative injury while bridging to allotransplantation. Low preformed antibody levels and immune plasticity in infancy provide a rationale for study.	Predicting an unacceptable palliative outcome is difficult. Graft size, growth, durability, and neonatal immunosuppression remain uncertain. Potential immunological advantages are unproven clinically. Parents must authorize substantial long-term risks before the child can participate.
**After Stage I, before Stage II palliation** Infants with a failing interstage course who cannot safely reach Stage II and lack an adequate bridge.	Could provide rescue support during a failing interstage course. May avoid further palliation while bridging to allotransplantation.	Redo sternotomy and altered arch, pulmonary-artery, or shunt anatomy complicate implantation. Rapid deterioration may limit assessment and preparation. Prior exposures require individual antibody evaluation; surgical history does not establish anti-pig sensitization. Effective established MCS remains a comparator.
**After Stage II, before Fontan** Children with failing Glenn physiology or unsuitable Fontan candidacy who meet transplant criteria and lack an adequate bridge.	Could avoid Fontan completion in a child with a poor expected outcome. May provide replacement circulation before further systemic venous congestion and extracardiac injury.	Assess VAD eligibility, feasibility, and expected bridge performance. Collaterals, vascular abnormalities, and prior operations increase operative complexity. Donor-specific compatibility testing remains necessary.
**After Fontan completion** Transplant-eligible patients with failing Fontan physiology and inadequate conventional options, including selected patients with ventricular or lymphatic complications.	Could provide a bridge when a human donor is unavailable and effective MCS is not feasible. Sustained graft function might relieve venous congestion and reversible complications, as observed after allotransplantation.	Assess correctable hemodynamic abnormalities, targeted lymphatic treatments, and VAD suitability for the mechanism of Fontan failure. Operative reconstruction, collaterals, and bleeding may be substantial. Advanced hepatic decompensation may require heart-liver transplantation and limit benefit from an isolated cardiac graft.

Potential advantages are potential, not established pediatric clinical outcomes. At every stage, multidisciplinary assessment should include anatomy, organ function, neurological prognosis, infection risk, donor-specific antibodies and crossmatch, family authorization, and long-term follow-up. Selection should also consider the anticipated bridge duration, the likelihood of timely allograft availability, and feasible options if xenograft function deteriorates before allotransplantation. Shared risks include rejection, thrombosis, immunosuppressive toxicity, donor-derived infection, and uncertain effects on later allotransplantation. Lifelong infection surveillance may remain necessary after graft removal. GE, gene-edited; MCS, mechanical circulatory support; VAD, ventricular assist device.

.

Before Stage I palliation, the principal potential advantage of cardiac xenotransplantation is immediate access to a size-appropriate graft for neonates who are unlikely to survive Stage I palliation or the allograft waitlist and who lack a durable mechanical bridge. In this setting, a xenograft would replace the initial palliative pathway and provide a direct bridge to allotransplantation. The threshold for such an approach would necessarily be high: contemporary staged palliation provides acceptable long-term outcomes for many neonates, and an investigational xenograft would need to offer a reasonable prospect of benefit over established therapy. Therefore, current expert proposals have focused on highly selected infants with major risk factors for Stage I palliation, such as atrioventricular valve dysfunction, unfavorable pulmonary artery anatomy, and ventricular dysfunction, rather than routine primary xenotransplantation for single-ventricle disease (6, 7).

After Stage I palliation, xenotransplantation becomes a potential rescue strategy for infants with a failing interstage course who cannot progress safely to Stage II palliation. Many contraindications or risk factors for progression to Stage II would not necessarily limit the benefit of cardiac xenotransplantation, including atrioventricular valve dysfunction and ventricular dysfunction. The indication would be strongest when an allograft is unavailable and MCS cannot provide a durable bridge. This remains a difficult population for mechanical support: small body size and single-ventricle physiology are associated with high waitlist risk, and the survival benefit associated with ventricular assist device (VAD) support in other pediatric transplant candidates has not been consistently observed in patients with single-ventricle disease (47). Prior Stage I palliation should not itself exclude xenotransplantation, although anti-pig antibody testing and donor-specific compatibility assessment would be required before treatment (34, 48).

After Stage II palliation, heart transplantation may be indicated when Glenn physiology is failing or Fontan completion is no longer appropriate. VAD support is a realistic bridge at this stage. In a 15-center Advanced Cardiac Therapies Improving Outcomes Network cohort that included 34 children with failed Stage II palliation (median age, 1.8 years; median weight, 10 kg), 24 patients reached transplantation and one recovered during a median support period of 74 days, although adverse events occurred in 25 patients (74%), including central nervous system events in 10 (29%) (49). As body size and anatomy permit the use of an implantable VAD, the possibility of prolonged support and rehabilitation at home provides a stronger conventional alternative and makes the rationale for xenotransplantation less compelling (50, 51).

After Fontan completion, transplantation may relieve protein-losing enteropathy, plastic bronchitis, and hepatic congestion, providing a physiologic basis for considering similar benefits from a functioning xenograft (52–54). Treatment selection must also account for correctable hemodynamic abnormalities, targeted lymphatic interventions, and the suitability of VAD support for the mechanism of Fontan failure (55, 56). Across both stages, the potential role of xenotransplantation would therefore be greatest when an allograft is unavailable and medical or mechanical support cannot provide an adequate bridge. Expected benefit would depend on sustained xenograft function and the reversibility of extracardiac disease; advanced hepatic decompensation may require combined heart–liver transplantation and limit the benefit of an isolated cardiac xenograft (57).

Across all stages, assessment should also weigh the anticipated waiting time for a suitable human allograft against the uncertain duration of reliable xenograft support. Recipient size, blood-group compatibility, sensitization, and donor availability would inform the likelihood of obtaining an allograft within that period; a prolonged or unpredictable wait would weaken the rationale for a bridge strategy. A contingency plan should address xenograft deterioration before an allograft becomes available, including treatment of reversible causes and reassessment of the feasibility of temporary MCS. If neither xenograft recovery nor further bridging is feasible, goals of care would need to be revisited with the family. These remain unresolved selection and management questions rather than validated criteria.

## 5 Ethical considerations for pediatric cardiac xenotransplantation

In 1984, Baby Fae, a neonate with hypoplastic left heart syndrome, received a baboon heart and survived for 20 days. The case prompted debate about the evidence required before clinical translation, parental permission, and the use of animals as organ sources. These concerns remain relevant to contemporary pediatric cardiac xenotransplantation, despite substantial changes in donor genetic engineering and immunosuppression. The central ethical question is under what circumstances the anticipated benefit to a child justifies the uncertainties and burdens of an investigational transplant (58, 59).

The timing of pediatric clinical trials remains debated. An adult-first approach emphasizes children's vulnerability and the need to establish safety and benefit in recipients able to provide informed consent. Others argue that children warrant independent consideration because their clinical alternatives, physiology, and potential immunological advantages differ from those of adults (60, 61). The 2026 International Society for Heart and Lung Transplantation consensus identifies selected infants with complex CHD as potential candidates but does not resolve the ethical justification for their enrollment. Assessment should therefore compare anticipated benefit with the realistic prospects of further palliation, mechanical support, and timely allotransplantation. When xenotransplantation is proposed as a bridge, continued allotransplant eligibility and options following xenograft failure must also be considered (38, 62).

Human decedent studies have been proposed as an additional means of addressing uncertainties before exposing living recipients to xenotransplantation. Age-appropriate nonhuman-primate studies remain essential, but species differences in anti-pig antibody reactivity can limit their predictive value for human recipients (63). Proponents emphasize the opportunity to examine these interactions directly in humans; critics question the additional information gained and caution that brain death-associated inflammation, endocrine disturbance, and hemodynamic instability may confound graft assessment (64–66). The surgical configuration also determines the questions that can be answered: a non-load-bearing heterotopic graft cannot establish the capacity for circulatory support assessed by orthotopic transplantation. These approaches entail different anatomical and perioperative requirements, particularly in small recipients.

Investigating durable immune response or graft growth over time in a decedent study would require prolonged observation, which could impose substantial emotional and practical burdens on bereaved families through continued somatic support of their deceased child. Shorter studies may lessen these burdens but restrict the questions that can be addressed, reinforcing concerns about the additional information these experiments can provide. The ethical assessment of pediatric decedent research must therefore consider whether the knowledge reasonably expected from extending observation justifies the associated burdens. Family preferences, ongoing support, and agreed limits on study duration and criteria for early termination would be central to this assessment (65, 67).

The role of pediatric decedent studies consequently remains unsettled. The National Heart, Lung, and Blood Institute workshop recognized their potential contribution while identifying unresolved questions concerning authorization, oversight, and research duration. Any pediatric proposal would require a specific scientific rationale, assessment of participant availability and technical feasibility, independent ethical review, and explicit parental authorization. Protocols should also protect opportunities for clinical organ donation. Neither the potential scientific value nor the limitations of this approach alone settle whether a particular study is justified (67).

For living pediatric recipients, parental permission should reflect an understanding of uncertain benefit, graft failure, immunosuppressive toxicity, and donor-derived infection. Initial candidates should undergo independent multidisciplinary review of eligibility and available alternatives, like the external review undertaken before the first compassionate-use cardiac xenotransplantation (11). Families should also have access to counselling from professionals independent of the investigational team. Discussions should distinguish research objectives from the prospect of benefit to the child and address alternatives without overstating likely success. Permission should be an ongoing process, supplemented by assent as development permits and the recipient's own consent for continued research upon reaching the legal age of consent (68, 69).

Long-term infection surveillance creates obligations extending beyond the initial transplant. U.S. Food and Drug Administration guidance recommends lifelong monitoring, and subsequent xenograft removal does not necessarily eliminate this need (68, 70). Families and contacts with repeated exposure involving exchange of the recipient's body fluids, including blood or saliva, should receive counselling about potential risks of zoonotic infection and appropriate precaution. This counselling should include FDA recommendations for deferral from blood or tissue donation by recipients and such contacts (70). Proposals to secure future adherence through a Ulysses contract remain contested, particularly when parents authorize participation on a child's behalf (71). Protocols should distinguish withdrawal from research from continuing clinical and public health responsibilities, explain how later disagreement will be addressed, and support follow-up through transition to adult care. Before enrollment, the study sponsor and transplant center should establish a funded plan assigning responsibility for lifelong surveillance and practical support. The transplant center should designate a team to coordinate follow-up and transfer to adult services, with arrangements to maintain support despite changes in responsible clinicians, trial closure, or sponsor withdrawal. These arrangements should protect privacy and provide practical assistance so that participation does not depend solely on a family's resources (68–70).

## 6 Conclusion

Pediatric cardiac xenotransplantation may provide a bridge to allotransplantation for selected children with a poor prognosis under available treatment. Candidate selection should account for alternatives to transplantation, extracardiac disease, and continued allotransplant eligibility. Preclinical findings support further investigation, but clinical benefit and long-term safety remain uncertain. Clinical use will require sustained graft function, appropriate graft growth, tolerable immunosuppression, and plans for graft failure and subsequent treatment. Parental permission, developing patient autonomy, and lifelong infection surveillance must also be addressed.

## References

[1] Cohen W Combs P El-Zein C Ilbawi M Kim G Jeevanandam V. Outcomes from three decades of infant and pediatric heart transplantation. ASAIO J. (2021) 67:1051–9. 10.1097/mat.0000000000001312 33181541

[2] Butto A Wright LK Dyal J Mao CY Garcia R Mahle WT. Impact of ventricular assist device use on pediatric heart transplant waitlist mortality: analysis of the scientific registry of transplant recipients database. Pediatr Transplant. (2024) 28:e14787. 10.1111/petr.14787 38766980

[3] Colvin MM Smith JM Stomberski CT Ahn YS Lindblad KA Messick EH. OPTN/SRTR 2024 annual data report: heart. Am J Transplant. (2026) 26:S367–473. 10.1016/j.ajt.2026.05.723 42276700

[4] Singh TP Cherikh WS Hsich E Harhay MO Hayes D Perch M. The international thoracic organ transplant registry of the international society for heart and lung transplantation: twenty-fifth pediatric heart transplantation report-2022; focus on infant heart transplantation. J Heart Lung Transplant. (2022) 41:1357–65. 10.1016/j.healun.2022.07.019 35989143 PMC10281815

[5] Conway J Cantor R Koehl D Spicer R Gupta D McCulloch M. Survival after heart transplant listing for infants on mechanical circulatory support. J Am Heart Assoc. (2020) 9:e011890. 10.1161/jaha.118.011890 33076747 PMC7763397

[6] Konstantinov IE Cooper DKC Adachi I Bacha E Bleiweis MS Chinnock R. Consensus statement on heart xenotransplantation in children: toward clinical translation. J Thorac Cardiovasc Surg. (2023) 166:960–7. 10.1016/j.jtcvs.2022.09.001 36184321 PMC10124772

[7] Cleveland JD Mitchell C Cooper DKC Cleveland DC. The potential of cardiac xenotransplantation for management of infants with complex congenital heart disease. Transl Pediatr. (2023) 12:2247–55. 10.21037/tp-22-664 38197100 PMC10772834

[8] Kawai T Williams WW Elias N Fishman JA Crisalli K Longchamp A. Xenotransplantation of a porcine kidney for end-stage kidney disease. N Engl J Med. (2025) 392:1933–40. 10.1056/nejmoa2412747 39927618

[9] United Therapeutics. Study to evaluate the safety and efficacy of the 10 GE xenokidney in patients with ESRD (EXPAND). ClinicalTrials.gov, NCT06878560 (2025). Accessed March 27, 2026. Available online at: https://clinicaltrials.gov/study/NCT06878560 (Accessed September 12, 2026).

[10] United Therapeutics. United Therapeutics Corporation announces FDA clearance of its Investigational New Drug Application for the UKidney Xenotransplantation Clinical Trial (2025). Available online at: https://ir.unither.com/press-releases/2025/02-03-2025-120011819 (Accessed September 12, 2026).

[11] Griffith BP Goerlich CE Singh AK Rothblatt M Lau CL Shah A. Genetically modified porcine-to-human cardiac xenotransplantation. N Engl J Med. (2022) 387:35–44. 10.1056/nejmoa2201422 35731912 PMC10361070

[12] Mohiuddin MM Singh AK Scobie L Goerlich CE Grazioli A Saharia K. Graft dysfunction in compassionate use of genetically engineered pig-to-human cardiac xenotransplantation: a case report. Lancet. (2023) 402:397–410. 10.1016/s0140-6736(23)00775-4 37393920 PMC10552929

[13] Griffith BP Grazioli A Singh AK Tully A Galindo J Saharia KK. Transplantation of a genetically modified porcine heart into a live human. Nat Med. (2025) 31:589–98. 10.1038/s41591-024-03429-1 39779924

[14] Moazami N Stern JM Khalil K Kim JI Narula N Mangiola M. Pig-to-human heart xenotransplantation in two recently deceased human recipients. Nat Med. (2023) 29:1989–97. 10.1038/s41591-023-02471-9 37488288

[15] Cleveland JD Mitchell CB Swicord W Neal SJ Vo C Bakshi K. Gene-edited pig cardiac xenotransplantation as a bridge to allotransplantation in infants: progress in a pig-to-baboon model. Am J Transplant. (2026) 26:980–91. 10.1016/j.ajt.2025.12.017 41453738 PMC12935157

[16] Hurst DJ Padilla L Merlocco A Rodger D Bobier C Gray WH. Pediatric cardiac xenotransplantation: recommendations for the ethical design of clinical trials. Transplantation. (2024) 108:e292–300. 10.1097/tp.0000000000004968 38419158

[17] Goldberg CS Trachtenberg F William Gaynor J Mahle WT Ravishankar C Schwartz SM. Longitudinal follow-up of children with HLHS and association between Norwood shunt type and long-term outcomes: the SVR III study. Circulation. (2023) 148:1330–9. 10.1161/circulationaha.123.065192 37795623 PMC10589429

[18] Hill KD Kang L Wang Q Newburger JW Gaynor JW Burns KM. Single-ventricle disease: ong-term outcomes and global morbidity in the single ventricle reconstruction trial. J Am Coll Cardiol. (2026) 87:3114–33. 10.1016/j.jacc.2026.01.049 41811273 PMC12979933

[19] Ross HJ Law Y Book WM Broberg CS Burchill L Cecchin F. Transplantation and mechanical circulatory support in congenital heart disease: a scientific statement from the American Heart Association. Circulation. (2016) 133:802–20. 10.1161/cir.0000000000000353 26797468

[20] Jayakumar KA Addonizio LJ Kichuk-Chrisant MR Galantowicz ME Lamour JM Quaegebeur JM. Cardiac transplantation after the fontan or glenn procedure. J Am Coll Cardiol. (2004) 44:2065–72. 10.1016/j.jacc.2004.08.031 15542293

[21] Lee S-Y Kim S-J Lee C-H Park CS Choi ES Ko H. The long-term outcomes and risk factors of complications after fontan surgery: from the Korean fontan registry (KFR). Korean Circ J. (2024) 54:653–68. 10.4070/kcj.2023.0211 39175341 PMC11522783

[22] Kelly B Ravndal M Nielsen AKM Krogh E Hjortdal VE Idorn L. 30-year Survival after the fontan operation in Denmark. Pediatr Cardiol. (2026) 47:1229–37. 10.1007/s00246-025-03896-4 40404979 PMC12901087

[23] Poh CL Cordina RL Iyengar AJ Zannino D Grigg LE Wheaton GR. Pre- and post-operative determinants of transplantation-free survival after fontan. The Australia and New Zealand experience. Int J Cardiol Heart Vasc. (2021) 35:100825. 10.1016/j.ijcha.2021.100825 34286062 PMC8273214

[24] Guerrero-Chalela CE Therrien J Grossman Y Guo L Liu A Marelli A. Severe fontan-associated liver disease and its association with mortality. J Am Heart Assoc. (2023) 12:e024034. 10.1161/jaha.121.024034 37776221 PMC10727255

[25] Boyd RM Laternser C Migally K Patel SG Dababneh R Carr MR. Fontan-related lymphatic complications: incidence, risk factors, and impact on extracardiac \outcomes and morbidity. J Am Heart Assoc. (2026) 15:e043310. 10.1161/jaha.125.043310 42261941 PMC13323181

[26] Kulshrestha K Greenberg JW Kennedy JT Hogue S Winlaw DS Ashfaq A. The majority of pediatric fontan patients have excellent post-transplant survival. J Thorac Cardiovasc Surg. (2024) 167:2193–203. 10.1016/j.jtcvs.2023.09.044 37774778 PMC10965507

[27] John MM Razzouk AJ Chinnock RE Bock MJ Kuhn MA Martens TP. Primary transplantation for congenital heart disease in the neonatal period: long-term outcomes. Ann Thorac Surg. (2019) 108:1857–64. 10.1016/j.athoracsur.2019.06.022 31362016

[28] John MM Zinyandu T Rosenblum JM Shashidharan S Chai PJ Shaw FR. Neonatal heart transplantation in the United States: trends and outcomes. Pediatr Transplant. (2024) 28:e14792. 10.1111/petr.14792 38808741

[29] Richmond ME Conway J Kirklin JK Cantor RS Koehl DA Lal AK. Three decades of collaboration through the pediatric heart transplant society registry: a journey through registry data with a highlight on children with single ventricle anatomy. Pediatr Transplant. (2024) 28:e14615. 10.1111/petr.14615 37811686

[30] Mitchell CB Simmons J Hodo CL Neal SJ Chitta S Vo C. Early results of an infant model of orthotopic cardiac xenotransplantation. J Heart Lung Transplant. (2025) 44:503–10. 10.1016/j.healun.2024.12.011 39778609 PMC12025259

[31] Mitchell CB Simmons J Vo C Bakshi K Juliani J Fenske J. Preservation of cardiac xenografts in a model of infant human cardiac transplantation. Xenotransplantation. (2025) 32:e70009. 10.1111/xen.70009 39786348 PMC12054934

[32] Cooper DKC Habibabady Z Kinoshita K Hara H Pierson RN. The respective relevance of sensitization to alloantigens and xenoantigens in pig organ xenotransplantation. Hum Immunol. (2023) 84:18–26. 10.1016/j.humimm.2022.06.003 35817653 PMC10154072

[33] Rood PPM Tai H-C Hara H Long C Ezzelarab M Lin YJ. Late onset of development of natural anti-nonGal antibodies in infant humans and baboons: implications for xenotransplantation in infants. Transpl Int. (2007) 20:1050–8. 10.1111/j.1432-2277.2007.00546.x 17850234

[34] Li Q Hara H Banks CA Yamamoto T Ayares D Mauchley DC. Anti-pig antibody in infants: can a genetically engineered pig heart bridge to allotransplantation? Ann Thorac Surg. (2020) 109:1268–73. 10.1016/j.athoracsur.2019.08.061 31580857

[35] Fan X Ang A Pollock-BarZiv SM Dipchand AI Ruiz P Wilson G. Donor-specific B-cell tolerance after ABO-incompatible infant heart transplantation. Nat Med. (2004) 10:1227–33. 10.1038/nm1126 15502841

[36] Urschel S Ballweg JA Cantor RS Koehl DA Reinhardt Z Zuckerman WA. Clinical outcomes of children receiving ABO-incompatible versus ABO-compatible heart transplantation: a multicentre cohort study. Lancet Child Adolesc Health. (2021) 5:341–9. 10.1016/s2352-4642(21)00023-7 33743201

[37] Goerlich CE Griffith B Hanna P Hong SN Ayares D Singh AK. The growth of xenotransplanted hearts can be reduced with growth hormone receptor knockout pig donors. J Thorac Cardiovasc Surg. (2023) 165:e69–81. 10.1016/j.jtcvs.2021.07.051 34579956 PMC8894505

[38] Mehra MR Mohiuddin MM Reichart B Abicht J-M Boulet J Cooper DKC. The 2026 international society for heart and lung transplantation consensus statement on clinical cardiac xenotransplantation. J Heart Lung Transplant. (2026) 45:e129–47. 10.1016/j.healun.2025.12.012 41718516

[39] Isaacson E Lucjak C Johnson WK Yin Z Wang T Rein L. Mitral valve surgery in neonates, infants, and children: surgical approach, outcomes, and predictors. Semin Thorac Cardiovasc Surg. (2020) 32:541–50. 10.1053/j.semtcvs.2020.01.005 31972302

[40] Rajab TK. Evidence-based surgical hypothesis: partial heart transplantation can deliver growing valve implants for congenital cardiac surgery. Surgery. (2021) 169:983–5. 10.1016/j.surg.2020.07.051 32948337

[41] Turek JW Kang L Overbey DM Carboni MP Rajab TK. Partial heart transplant in a neonate with irreparable truncal valve dysfunction. JAMA. (2024) 331:60–4. 10.1001/jama.2023.23823 38165407 PMC10762570

[42] Masri A Shi CY Winemiller B Alsarrawi H Kochilas LK Reemtsen B. Short- and long-term outcomes of systemic semilunar valve replacement in neonates and infants. NPJ Cardiovasc Health. (2026) 3:12. 10.1038/s44325-026-00109-6 41872460 PMC13009365

[43] Kassier AB Masri A Iqfat H Zaghw A Shi CY Kochilas LK. Long-term outcomes of pulmonary valve replacement in infancy: time for a reassessment? Prog Pediatr Cardiol. (2026) 80:101901. 10.1016/j.ppedcard.2025.101901 42318134 PMC13274725

[44] Overbey DM Aykut B Kucera JA Medina CK Sethi NJ Barker PCA. Partial heart transplant for congenital heart disease. JAMA. (2025) 334:1077–83. 10.1001/jama.2025.13580 40864436 PMC12457970

[45] Hill MA Kwon JH Gerry B Hardy WA Walkowiak OA Kavarana MN. Immune privilege of heart valves. Front Immunol. (2021) 12:731361. 10.3389/fimmu.2021.731361 34447390 PMC8383064

[46] Mitchell CB Wiggins LM Wells WJ Cleveland DC Cleveland JD. A novel model for xenograft right ventricle to pulmonary artery conduit. ASAIO J. (2024) 70:e139–41. 10.1097/mat.0000000000002213 38669640

[47] Butts RJ Toombs L Kirklin JK Schumacher KR Conway J West SC. Waitlist outcomes for pediatric heart transplantation in the current era: an analysis of the pediatric heart transplant society database. Circulation. (2024) 150:362–73. 10.1161/circulationaha.123.068189 38939965

[48] Oscherwitz M Nguyen HQ Raza SS Cleveland DC Padilla LA Sorabella RA. Will previous palliative surgery for congenital heart disease be detrimental to subsequent pig heart xenotransplantation? Transpl Immunol. (2022) 74:101661. 10.1016/j.trim.2022.101661 35787933 PMC9762890

[49] Rabinowitz EJ Mehegan M Joong A Shezad M Lorts A Villa CR. Multicenter outcomes for ventricular assist device support for failed stage II palliation. JHLT Open. (2024) 3:100015. 10.1016/j.jhlto.2023.100015 40145104 PMC11935379

[50] O’Connor MJ Shezad M Ahmed H Amdani S Auerbach SR Bearl DW. Expanding use of the HeartMate 3 ventricular assist device in pediatric and adult patients within the advanced cardiac therapies improving outcomes network (ACTION). J Heart Lung Transplant. (2023) 42:1546–56. 10.1016/j.healun.2023.06.015 37419295

[51] Schweiger M Hussein H De By TMMH Zimpfer D Sliwka J Davies B. Use of intracorporeal durable LVAD support in children using HVAD or HeartMate 3-A EUROMACS analysis. J Cardiovasc Dev Dis. (2023) 10:351. 10.3390/jcdd10080351 37623364 PMC10455245

[52] Schumacher KR Yu S Butts R Castleberry C Chen S Edens E. Fontan-associated protein-losing enteropathy and post‒heart transplant outcomes: a multicenter study. J Heart Lung Transplant. (2019) 38:17–25. 10.1016/j.healun.2018.09.024 30391195

[53] Gossett JG Almond CS Kirk R Zangwill S Richmond ME Kantor PF. Outcomes of cardiac transplantation in single-ventricle patients with plastic bronchitis: a multicenter study. J Am Coll Cardiol. (2013) 61:985–6. 10.1016/j.jacc.2012.10.042 23290545

[54] Griffiths ER Lambert LM Ou Z Shaaban A Rezvani M Carlo WF. Fontan-associated liver disease after heart transplant. Pediatr Transplant. (2023) 27:e14435. 10.1111/petr.14435 36380561

[55] Dori Y Keller MS Rome JJ Gillespie MJ Glatz AC Dodds K. Percutaneous lymphatic embolization of abnormal pulmonary lymphatic flow as treatment of plastic bronchitis in patients with congenital heart disease. Circulation. (2016) 133:1160–70. 10.1161/circulationaha.115.019710 26864093

[56] Chen S Simpson KE. ACTION learning network investigators. Over 100 fontan patients on systemic ventricular assist device support: an ACTION update. ASAIO J. (2025) 71:588–95. 10.1097/mat.0000000000002367 39723978

[57] Téllez L Payancé A Tjwa E Del Cerro MJ Idorn L Ovroutski S. EASL-ERN position paper on liver involvement in patients with fontan-type circulation. J Hepatol. (2023) 79:1270–301. 10.1016/j.jhep.2023.07.013 37863545

[58] Bailey LL Nehlsen-Cannarella SL Concepcion W Jolley WB. Baboon-to-human cardiac xenotransplantation in a neonate. JAMA. (1985) 254:3321–9. 10.1001/jama.1985.03360230053022 2933538

[59] Caplan AL. Ethical issues raised by research involving xenografts. JAMA. (1985) 254:3339–43. 10.1001/jama.1985.03360230071026 3906158

[60] Kögel J Schmoeckel M Marckmann G. Who shall go first? A multicriteria approach to patient selection for first clinical trials of cardiac xenotransplantation. J Med Ethics. (2025) 51:156–62. 10.1136/jme-2024-110056 39244213

[61] Hurst DJ Merlocco A Padilla LA Bobier C. Argument for allowing first-in-human cardiac xenotransplantation clinical trials in paediatric patients. J Med Ethics. (2025) 51:168–9. 10.1136/jme-2024-110452 39578045

[62] Fedson S Lavee J Bryce K Egan T Olland A Kanwar M. Ethical considerations in xenotransplantation of thoracic organs - a call for a debate on value based decisions. J Heart Lung Transplant. (2024) 43:1033–8. 10.1016/j.healun.2024.03.012 38775760

[63] Takemoto S Burdorf L Pierson RN 3rd. Pig lung xenotransplantation: barriers on the road to clinical translation. Transpl Int. (2026) 38:15542. 10.3389/ti.2025.15542 41583874 PMC12827209

[64] Montgomery RA Griesemer AD Segev DL Sommer P. The decedent model: a new paradigm for de-risking high stakes clinical trials like xenotransplantation. Am J Transplant. (2024) 24:526–32. 10.1016/j.ajt.2024.01.035 38341026

[65] Cooper DKC Kobayashi T. Xenotransplantation experiments in brain-dead human subjects-A critical appraisal. Am J Transplant. (2024) 24:520–5. 10.1016/j.ajt.2023.12.020 38158188 PMC13020695

[66] Cooper DKC Mou L Cleveland JD Simmons JH Cleveland DC. Xenotransplantation research -the nonhuman primate model is preferable to the human decedent model. Transpl Int. (2025) 38:14452. 10.3389/ti.2025.14452 40852500 PMC12367563

[67] Khush KK Bernat JL Pierson RN Silverman HJ Parent B Glazier AK. Research opportunities and ethical considerations for heart and lung xenotransplantation research: a report from the national heart, lung, and blood institute workshop. Am J Transplant. (2024) 24:918–27. 10.1016/j.ajt.2024.03.015 38514013 PMC11144553

[68] Hurst DJ Bobier C Merlocco A Padilla LA Rodger D Cleveland D. Pediatric cardiac xenotransplantation and expanded access: ethical considerations. Pediatr Transplant. (2024) 28:e14876. 10.1111/petr.14876 39412392

[69] Hawthorne WJ Wolf E Cowan PJ Wang W Yun IJ Brenner P. Global consultation for clinical xenotransplantation: international xenotransplantation association consensus and communiqué update. Xenotransplantation. (2026) 33:e70157. 10.1111/xen.70157 42548012 PMC13433974

[70] U.S. Food and Drug Administration. Source Animal, Product, Preclinical, and Clinical Issues Concerning the Use of Xenotransplantation Products in Humans: Guidance for Industry. Silver Spring, MD: U.S. Food and Drug Administration (2016). Available online at: https://www.fda.gov/media/102126/download (Accessed September 12, 2026).

[71] Hurst DJ Padilla L Schiff T Parent B. Revisiting the use of ulysses contracts in xenotransplantation. Transplantation. (2024) 108:369–73. 10.1097/tp.0000000000004679 37246302

